# MR-Imaging and Histopathological Diagnostic Work-Up of Patients with Spontaneous Lobar Intracerebral Hemorrhage: Results of an Institutional Prospective Registry Study

**DOI:** 10.3390/diagnostics11020368

**Published:** 2021-02-22

**Authors:** Patrick Schuss, Christian Bode, Valeri Borger, Christoph Coch, Ági Güresir, Alexis Hadjiathanasiou, Motaz Hamed, Klaus Kuchelmeister, Felix Lehmann, Marcus Müller, Matthias Schneider, László Solymosi, Hartmut Vatter, Markus Velten, Erdem Güresir

**Affiliations:** 1 Department of Neurosurgery, University Hospital Bonn, Venusberg-Campus 1, 53127 Bonn, Germany; valeri.borger@ukbonn.de (V.B.); agi.gueresir@ukbonn.de (Á.G.); alexis.hadjiathanasiou@ukbonn.de (A.H.); motaz.hamed@ukbonn.de (M.H.); matthias.schneider@ukbonn.de (M.S.); hartmut.vatter@ukbonn.de (H.V.); erdem.gueresir@ukbonn.de (E.G.); 2 Department of Anesthesiology and Intensive Care Medicine, University Hospital Bonn, Venusberg-Campus 1, 53127 Bonn, Germany; christian.bode@ukbonn.de (C.B.); felix.lehmann@ukbonn.de (F.L.); markus.velten@ukbonn.de (M.V.); 3 Study Center Bonn (SZB), Clinical Study Core Unit, University Hospital Bonn, Venusberg-Campus 1, 53127 Bonn, Germany; ccoch@uni-bonn.de; 4 Institute of Neuropathology, University Hospital Bonn, Venusberg-Campus 1, 53127 Bonn, Germany; klaus.kuchelmeister@ukbonn.de; 5 Department of Neurology, University Hospital Bonn, Venusberg-Campus 1, 53127 Bonn, Germany; marcus_mathias.mueller@ukbonn.de; 6 Department of Neuroradiology, University Hospital Bonn, Venusberg-Campus 1, 53127 Bonn, Germany; laszlo.solymosi@ukbonn.de

**Keywords:** intracerebral hemorrhage, etiology, diagnostic work-up, tumor, CAA, AVM

## Abstract

Intracerebral hemorrhage (ICH) is a frequently disabling or fatal disease. The localization of ICH often allows an etiological association. However, in atypical/lobar ICH, the cause of bleeding is less obvious. Therefore, we present prospective histopathological and radiological studies which were conducted within the diagnostic workup to identify causes for lobar ICH other than hypertension. From 2016 to 2018, 198 patients with spontaneous, non-traumatic ICH requiring neurosurgical monitoring were enrolled in an institutional prospective patient registry. Patients with deep-seated ICH and/or hemorrhagically transformed cerebral infarcts were excluded from further analysis. Data to evaluate the source of bleeding based on histopathological and/or radiological workup were prospectively evaluated and analyzed. After applying the inclusion criteria and excluding patients with incomplete diagnostic workup, a total of 52 consecutive patients with lobar ICH were further analyzed. Macrovascular disease was detected in 14 patients with lobar ICH (27%). In 11 patients, diagnostic workup identified cerebral amyloid angiopathy-related ICH (21%). In addition, five patients with tumor-related ICH (10%) and six patients with ICH based on infectious pathologies (11%) were identified. In four patients, the cause of bleeding remained unknown despite extensive diagnostic workup (8%). The present prospective registry study demonstrates a higher probability to identify a cause of bleeding other than hypertension in patients with lobar ICH. Therefore, a thorough diagnostic work-up in patients with ICH is essential to accelerate treatment and further improve outcome or prevent rebleeding.

## 1. Introduction

Spontaneous, non-traumatic intracerebral hemorrhage (ICH) is accountable for about 10% of strokes in the western population [1]. In addition to the hemorrhage itself, the subsequent changes, among others, might impede the recovery of the affected patient. The sudden influx of blood into the brain parenchyma leads not only to mechanical damage, but also to secondary neuronal death. Moreover, toxic factors derived from the hematoma aggregate to cause an inflammatory response [2]. Here, several cellular targets have recently been discovered in animal experimental studies, underscoring efforts to therapeutically support reconvalescence in these severely affected patients [3,4]. In addition to the consequences of the hemorrhage itself, the further therapeutic/surgical approach is the subject of numerous efforts [5,6,7,8]. Regarding their localization, spontaneous supratentorial ICHs can be divided into deep-seated and lobar ICH [9]. Established etiologies for ICH are long-lasting hypertension and cerebral amyloid angiopathy (CAA) [10,11]. Regarding their localization, spontaneous supratentorial ICHs can be divided into deep-seated and lobar ICH. Especially in patients with lobar/atypically located ICH, numerous other causes have been identified, including vascular malformations, intracranial aneurysms, and tumors [12,13,14]. In these cases, the timely identification of a potential cause of bleeding is mandatory in order to prevent secondary bleeding. Due to the multiple potential causes of lobar ICH and the considerable logistical and technical requirements, the optimal diagnostic evaluation remains debatable. In this prospective registry study, we present the observations made during the institutional diagnostic work-up of patients with lobar ICHs using histopathological and radiological studies.

## 2. Materials and Methods

### 2.1. ICH Registry Study

This prospective cohort study is based on the ICH registry of the Department of Neurosurgery, University of Bonn (DRKS00011098). The ICH registry is not population-based and participation is open to all hospitals treating patients with ICH in the region. All consecutive patients with spontaneous ICH who were admitted to the neurosurgical department during the study period were included in the present analysis. Patients were admitted due to lobar location of ICH or to deep-seated ICH, which endorsed further diagnostic clarification and a potential neurosurgical intervention. Patients with trauma-associated ICH or a hemorrhagic transformation of a cerebral infarction were excluded. Study data were prospectively collected and managed using REDCap electronic data capture tools hosted at the Study Center Bonn (SZB) [15]. Informed consent was obtained from the patient or patients’ representative. Information collected for each patient included sociodemographic characteristics, comorbidities, ICH location, ICH volume, diagnostic procedures, admission procedures, and treatment strategies during hospitalization. The study was approved by the local ethics committee (approval number 384/15) in accordance with the Declaration of Helsinki and registered in the German register of clinical trials (DRKS00011098).

### 2.2. Radiological Study

ICH was initially diagnosed with computed tomography (CT) or magnetic resonance imaging (MRI). In all admitted cases, additional CT-angiography (CTA) was performed. Patients with ICH were dichotomized into two groups according to the location of ICH. Patients with deep-seated supratentorial or infratentorial ICH (located in the basal ganglia, internal or external capsule or thalamus without extension to the lobar area, brainstem) were classified as patients with non-lobar ICH [16]. All other locations of ICH were defined as lobar ICH. Patients were admitted to our neurosurgical department due to the need for further diagnostic workup for unknown bleeding source, further prognostic evaluation, and the potential necessity of a neurosurgical intervention during treatment course. This was sought in space-occupying supratentorial deep-seated ICH, lobar ICH, involvement of the brainstem, and space-occupying infratentorial located ICH. Patients with no history of hypertension and lobar location of ICH underwent an additional MRI scan in order to identify a potential secondary cause of ICH. Cerebral MRI scans were obtained on a 3-Tesla GE Discovery MR750w scanner equipped with cardiac-enhanced gradients (40 mT/m). The following sequences were routinely obtained: T1-(sagittal) and T2-weighted imaging (axial), T1-weighted imaging with contrast, T2*-weighted gradient echo MRI, diffusion-weighted imaging (DWI), fluid-attenuated inversion recovery and 3-dimensional time-of-flight (TOF) magnetic resonance angiography of the circle of Willis. Three-dimensional TOF magnetic resonance venography was obtained in some patients. The volume of ICH was measured using the ABC/2-method [17].

### 2.3. Treatment of ICH

All patients received the best medical treatment according to the American Heart Association/American Stroke Association guidelines [18]. In patients with ICH and known anticoagulant usage prior to hospitalization, urgent reversal was carried out as described before [19]. In cases with persistent and treatment refractory intracranial pressure values >20 mmHg, decompressive hemicraniectomy (DC) with or without ICH evacuation was performed [8]. In patients with cerebellar ICH, suboccipital DC with or without ICH evacuation was performed if neurological deterioration and/or radiological signs of acute hydrocephalus occurred [20]. In subsequent treatment of the pathologies underlying the lobar ICH, the established guidelines were followed as previously reported [12,21,22].

### 2.4. Histopathological Study

In patients with lobar ICH and surgical hematoma evacuation, specimens were obtained intraoperatively for histopathological examination. The surgically removed tissue was routinely processed for paraffin histology. Four-micrometer thick paraffin sections were stained with hematoxylin and eosin (H&E). Depending on differential diagnosis, additional conventional (e.g., Congo red, Prussian blue) and immunohistochemical (using a Ventana BenchMark XT immunostainer (Ventana Medical Systems, Tuscon, AZ, USA) with various commercially available antibodies, e.g., antibodies against ß-amyloid) stains were prepared. The complete tissue of all the slides was evaluated under a binocular transmitted light microscope with various magnifications (ocular lens 10× and objective lenses at least 4×, 10×, 20×, 40×). Photos of representative histological areas were taken using an Olympus BX51 microscope with an Olympus DP70 digital camera system (Olympus America, Center Valley, PA, USA). To reduce background signals, a slide area without tissue was photographed and saved as background. The background of the photos with representative histology could automatically be corrected against this saved background photo.

### 2.5. Inclusion Criteria

**Figure 1:**
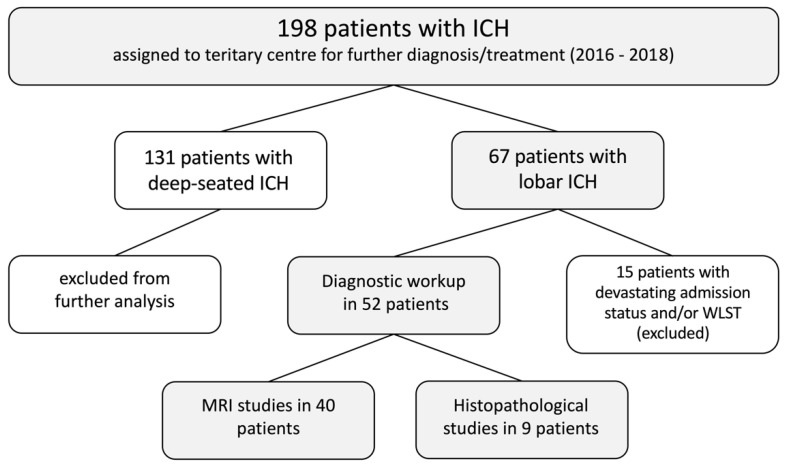
Patient enrollment. ICH = intracerebral hemorrhage; WLST = withdrawal of life-sustaining therapy; MRI = magnetic resonance imaging.

The present study was designed in order to investigate potential underlying pathologies in patients suffering from lobar ICH. Therefore, 198 eligible patients with ICH were screened and only patients with lobar ICH were included in further analysis. Patients suffering from non-lobar, deep-seated ICH were excluded. In addition, all patients with cerebellar ICH were excluded. Details are given in Figure 1.

## 3. Results

During the observation period between January 2016 and September 2018, 67 patients suffering from spontaneous, lobar ICH were admitted to our neurosurgical department. In 15 patients (22%), diagnostic work-up was not completed due to devastating admission status and/or the existence of a “withholding of life-sustaining treatment” (WLST) order, so that these patients were excluded from further analysis. This resulted in a total study population of 52 patients with lobar ICH, who were enrolled in the above-mentioned diagnostic workup and prospectively entered in the observational database. The mean age of patients with lobar ICH was 61 ± 15 years. Prior use of anticoagulation/new oral anticoagulants or antiplatelet medication was present in 16 patients suffering from lobar ICH (31%). A total of 22 patients with spontaneous lobar ICH presented with the pre-existing condition of hypertension (42%), 6 patients with coronary artery disease (11%), and 7 patients with diabetes mellitus type 2 (13%).

Patient characteristics are shown in detail in Table 1.

### 3.1. Treatment of ICH

A total of 22 patients with lobar ICH underwent surgical treatment (42%). One patient underwent the insertion of external ventricular drainage (EVD) for the diversion of cerebrospinal fluid without further surgical procedures (2%). Three patients underwent stereotactic aspiration of the intracerebral hematoma (6%). Two patients underwent craniotomy with additional ICH evacuation (4%). A total of 10 patients underwent DC without additional ICH evacuation (19%). In another six patients, DC with additional hematoma evacuation was performed (11%).

### 3.2. Etiology of ICH

**Figure 2:**
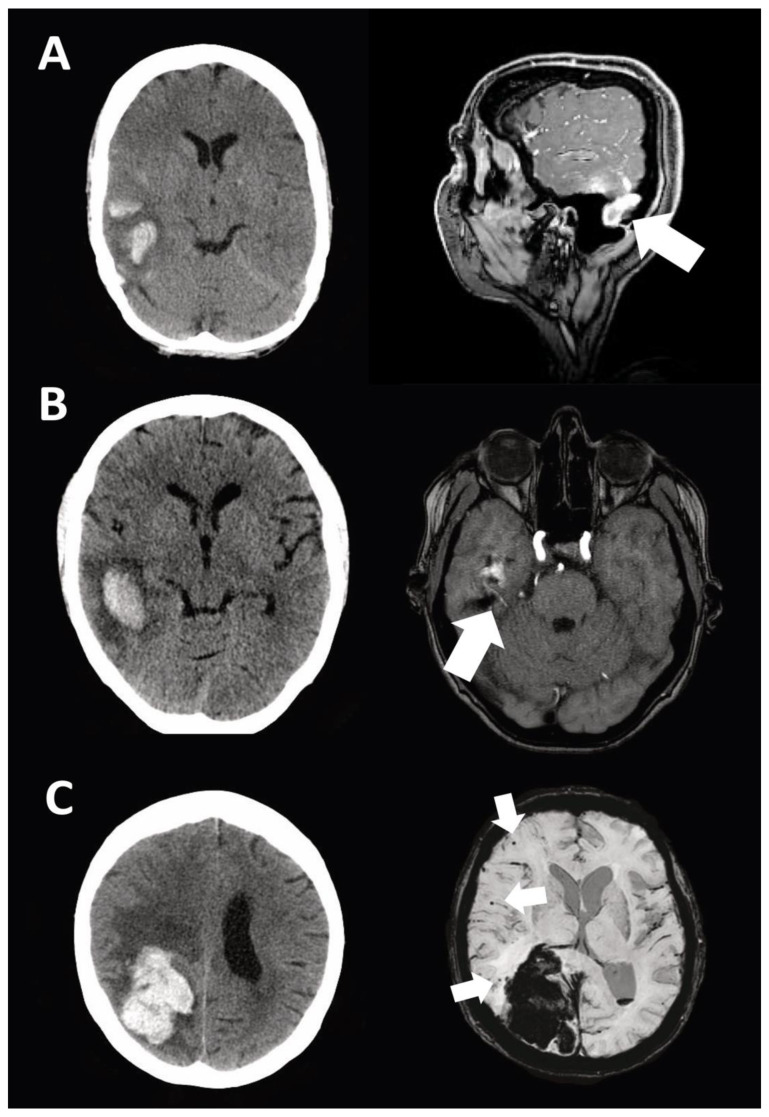
Exemplary cases with magnetic resonance imaging work-up for lobar ICH. (**A**) Lobar ICH caused by sinus venous thrombosis; (**B**) Lobar ICH caused by dural arteriovenous fistula; (**C**) Lobar ICH accompanied with underlying cerebral amyloid angiopathy (CAA). White arrow indicates the described pathology.

A total of 40 out of 52 patients (77%) suffering from spontaneous lobar ICH underwent further radiological evaluation using MRI (see exemplary case studies in Figure 2).

**Figure 3:**
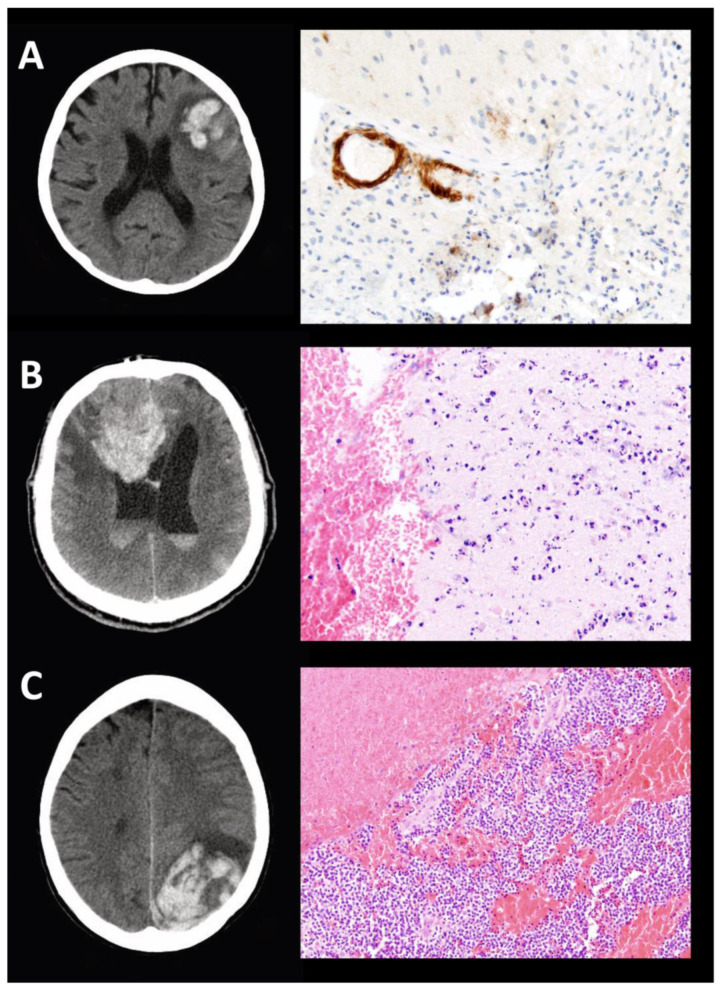
Exemplary cases with histopathological work-up for lobar ICH. (**A**) Lobar ICH accompanied with underlying CAA; (**B**) Lobar ICH accompanied with phlegmonic granulocytic infiltrates; (**C**) Lobar ICH caused by anaplastic oligodendroglioma; original magnification: A/B 200× (ocular lens 10×, objective lens 20×, NA 0.50), (C 100× (ocular lens 10×, objective lens 10×, NA 0.30).

In 9 out of 52 patients (17%) suffering from spontaneous lobar ICH, further histopathological investigation was performed after surgical biopsy or evacuation of ICH (see exemplary case studies in Figure 3).

Based on clinical, radiological and histopathological studies, the etiology of spontaneous lobar ICH was determined. Details on the determined etiology for lobar ICH are also given in Table 2. Intracranial macrovascular disease was identified as the underlying pathology for primary lobar ICH in a total of 14 patients (27%). In detail, seven patients presented with a ruptured intracranial aneurysm (13%), two patients with a ruptured arteriovenous malformation (4%), two patients with ruptured dural arteriovenous fistula (4%), two patients with acute sinus venous thrombosis (4%), and in one patient lobar ICH was caused by cerebral cavernous malformation (2%). In 11 patients, histopathological and/or radiological studies proved CAA-related lobar ICH (21%). Furthermore, in five patients, additional diagnostic workup revealed cancer as the underlying pathology for primary ICH (10%), with two patients suffering from glioblastoma (4%), one patient was diagnosed with oligodendroglioma (2%), and two suffering from intracranial metastases (4%). In all patients with a proven tumorous cause of lobar ICH, cancer was not discovered before the bleeding, and thus the resulting bleeding event led to the diagnosis of the tumor. In six patients, histopathological and/or radiological diagnostic workup revealed infectious embolic disease as the underlying pathology for lobar ICH (11%). In a total of six patients (11%), histopathological confirmation was obtained in the course of surgical ICH treatment or its source in addition to radiological workup (MRI). In one patient (2%), the preceding MRI could not conclusively pre-confirm (due to bleeding artifacts) the subsequently histologically verified diagnosis (glioblastoma). Additional digital subtraction angiography of intra- and extracranial vessels was conducted in 18 patients (35%). In four patients, the cause of the lobar ICH was considered unknown, even though extensive diagnostic workup was obtained (8%).

## 4. Discussion

The present analysis of the prospective patient cohort emphasizes that in patients with lobar located spontaneous intracerebral hemorrhage, comprehensive diagnostic workup may lead to the identification of the underlying cause of bleeding and the implementation of appropriate treatment.

ICH remains a form of cerebrovascular disorder associated with high mortality, despite all attempts at therapeutic and rehabilitative efforts [23,24]. Not only are primary hemorrhage-associated injuries of relevance, but secondary ICH-related injuries must also be taken into account [25]. For instance, the dysregulation of energy metabolism occurs in the setting of ICH, which could be a major contributor to secondary injury [26,27,28]. Furthermore, oxidative stress, inflammation, and iron toxicity also play important roles [2,29] [30]. However, in addition to the numerous pathophysiological mechanisms, it is also important to determine the underlying cause of the initial injury. Thus, it may be possible to prevent a repeat additive bleeding event.

Overall, hypertension remains the most common modifiable risk factor for ICH [31]. Hypertension-induced ICHs tend to be deep seated in the area of the basal ganglia and/or thalamus, and are therefore also commonly described as typically located ICH [32]. However, Lovelock et al. have previously reported a substantial decrease in hypertension-related ICH in recent decades [33]. Therefore, further diagnostic clarification in patients with ICH is crucial, especially if the location of the ICH is not deep-seated [32,34].

Cerebral amyloid angiopathy is also a well-established cause of primary ICH [35]. To improve the management of these patients, Knudsen et al. had established the Boston criteria for the clinical diagnosis of CAA, which were modified by the incorporation of superficial hemosiderin deposits [36,37]. The diagnosis of CAA is considered a challenge in daily clinical practice, as approximately 38% of patients with potential CAA according to the Boston criteria do not have pathological findings for CAA. The differentiation from exclusively hypertension-related ICH is usually based on the location of the bleeding (deep-seated versus lobar), but is nevertheless challenging due to the often imprecisely formulated definitions of hypertension-related ICH [38]. In the present series, the diagnosis of CAA was made in 21% of patients with lobar ICH. For the further diagnosis of an underlying CAA in the presence of lobar ICH, the implementation of an MRI scan is usually mandatory. Microbleeds detectable in the MRI scan increase the likelihood of a reliable diagnosis of CAA [39]. In vigilance-impaired patients with lobar ICH, the realization of an MRI scan can prove to be a challenge due to the sometimes necessary (intensive care) monitoring. However, the present study emphasizes the importance of adequate diagnostic workup, which should lead to a reasonable utilization of clinical and technical resources.

In the case of lobar ICH, other possible underlying pathologies have been described. These include arteriovenous malformations, intracranial aneurysms, infectious diseases, and even intracranial tumors. Since, in contrast to hypertension- and CAA-related ICH, the presence of one of the above-mentioned diseases as a source of bleeding results in a cascade of other possible treatment options, the lobar ICH represents an indication for further clarification. Jackson et al. reported that hypertension occurs twice as frequently in deep-seated ICH compared to lobar ICH [32]. As a consequence, there is high probability that the lobar ICH has a different cause. The present study emphasizes that patients with lobar ICH are more likely to suffer from an underlying pathology other than hypertension. However, patients with deep-seated ICH should not routinely be excluded from further diagnostic workup.

Nevertheless, early evaluation of an ICH is of tremendous importance, and yet subject to varying circumstances in its extent. Either the technical equipment or the existing/absent specialist expertise, but also patient-specific factors, can lead to underdiagnosis and/or overdiagnosis [40,41,42]. Thus, the establishment of universal, consistent diagnostic algorithms is difficult due to the individual situations to be considered. Previous studies have helped to develop classification criteria in order to improve the diagnostic evaluation of patients suffering from ICH [38,41]. For instance, the DIAGRAM prediction score might help to determine the probability of an underlying macrovascular cause in ICH [13]. Various neoplasms in the brain are reported to be prone to bleeding, such as glioblastoma and metastases from, e.g., melanoma, lung cancer, and/or renal cell cancer. The imaging features are highly variable due to the occurrence of often multiple bleedings at different times and potential concomitant necrosis and cyst formations. Therefore, the initial diagnosis of a potential tumorous cause of lobar ICH is not always attainable, particularly in extensive and large ICH. In addition, in cases with lobar ICH and unknown underlying pathology, MRI follow-up should be considered after 6–8 weeks to increase the likelihood of detecting potential contrast-enhancing lesions after the resolution of the bleeding, especially if a cancer history is known [14].

As a result of the thorough diagnostic work-up, only 8% of the patients remained unclear about the etiology of spontaneous lobar ICH in the present study. Yet, the rapid identification of the underlying pathology in patients with primary ICH potentially facilitates treatment opportunities and, subsequently, neurological and functional outcome [43]. In line with this, the results of the present prospective patient registry support the previously endorsed research priorities in patients with ICH [43].

The present study has several limitations. The patient cohort only includes patients with lobar ICH who were admitted for neurosurgical care. For this reason, selection bias must be assumed with the results of this study to be interpreted in the context that the patient selection did not constitute a representative ICH cohort. Furthermore, obtaining a histopathological diagnosis in most patients with lobar ICH was not feasible due to the risks and sometimes lack of subsequent treatment consequences when performing invasive brain biopsy. Due to the small number of cases, it was also not fully possible to weigh the advantages/disadvantages of the different confirmatory methods (histological versus radiological) against each other. Nevertheless, the results of the present prospective study mirror a structured diagnostic procedure of a single neurovascular center and should therefore be considered for further validation in future studies. Furthermore, the limitations of this study in particular should serve to foster a continued academic interest in the scientific processing of the different diagnostic algorithms.

## 5. Conclusions

Lobar intracerebral hemorrhage can be caused by several underlying pathologies. The present prospective patient cohort study demonstrates a higher probability to identify a cause of bleeding other than hypertension in patients with lobar ICH. Therefore, a thorough diagnostic workup in patients with lobar ICH is essential to accelerate the treatment of the cause of bleeding, and thus further improve outcome or prevent rebleeding.

## Figures and Tables

**Table 1:** Patients’ characteristics.

	Patients with Lobar ICH (*n* = 52)
Mean age (±SD; yrs)	61 ± 15
Female sex	28 (54%)
Anticoagulation medication prior ictus	5 (10%)
NOAC medication prior ictus	2 (4%)
Antiplatelet medication prior ictus	9 (17%)
**Comorbidities**	
Hypertension	22 (42%)
Coronary artery disease	6 (11%)
Diabetes mellitus	7 (13%)
**Treatment of ICH**	
Best medical treatment w/o any surgical therapy	30 (58%)
**Surgical treatment**	22 (42%)
EVD w/o further surgical procedures	1 (2%)
Stereotactic aspiration	3 (6%)
Craniotomy with ICH evacuation	2 (4%)
DC w/o ICH evacuation	10 (19%)
DC with ICH evacuation	6 (11%)

ICH = intracerebral hemorrhage; yrs = years; NOAC = new oral anticoagulant; w/o = without; EVD = external ventricular drainage; DC = decompressive craniectomy.

**Table 2:** Underlying pathology in patients with lobar ICH.

	Patients with Lobar ICH (*n* = 52)
**Macrovascular disease-related ICH**	**14** (**27%**)
Ruptured intracranial aneurysm	7 (13%)
Ruptured BAVM	2 (4%)
Ruptured DAVF	2 (4%)
Acute sinus venous thrombosis	2 (4%)
Cerebral cavernous malformation	1 (2%)
**CAA-related ICH**	**11** (**21%**)
**Tumor-related ICH**	**5** (**10%**)
Glioblastoma	2 (4%)
Oligodendroglioma	1 (2%)
Intracranial metastasis	2 (4%)
**Infectious embolic disease-related ICH**	**6** (**11%**)
**Unknown cause**	**4** (**8%**)

ICH = intracerebral hemorrhage; BAVM = brain arteriovenous malformation; DAVF = dural arteriovenous fistula; CAA = cerebral amyloid angiopathy. Bold text and associated numerical values reflect the total number of patients for the associated category.

## Data Availability

Data are available from the corresponding author upon reasonable request.
